# 1,2-Ethyl­enediaminium bis­(2-benzamido­benzoate)

**DOI:** 10.1107/S1600536813021028

**Published:** 2013-08-07

**Authors:** Mavlonbek Ziyaev, Jamshid Ashurov, Samat Talipov, Bakhtiyar Ibragimov

**Affiliations:** a Institute of Bioorganic Chemistry, Academy of Sciences of Uzbekistan, M. Ulugbek Str. 83, Tashkent 100125, Uzbekistan

## Abstract

In the title salt, C_2_H_10_N_2_^2+^·2C_14_H_10_NO_3_^−^, the ethyl­ene­diaminium dication lies on an inversion centre. In the anion, the benzene rings make a dihedral angle of 33.87 (9)° and intramolecular N—H⋯O and C—H⋯O hydrogen bonds occur. All the amino H atoms are involved in N—H⋯O hydrogen bonds. These hydrogen bonds link the ionic units into a three-dimensional network. In addition, the crystal structure also features weak C—H⋯O inter­actions.

## Related literature

For the crystal structure of 1,2-ethyl­enedi­ammonium salts of aromatic acids, see: Shen-Tu *et al.* (2008 ▶); Zhao & Feng (2011 ▶). For the crystal structure of the 2-benzamido­benzoyl acid Cu^II^ coordination compound, see: Kaizer *et al.* (2006 ▶). 
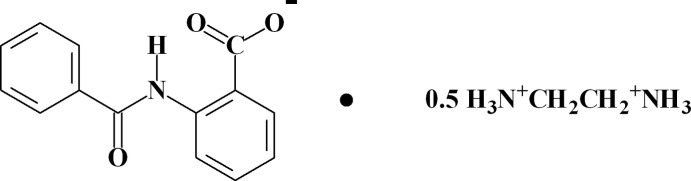


## Experimental

### 

#### Crystal data

0.5C_2_H_10_N_2_^2+^·C_14_H_10_NO_3_^−^
*M* *_r_* = 271.29
Monoclinic, 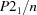

*a* = 5.314 (1) Å
*b* = 13.745 (2) Å
*c* = 18.580 (4) Å
β = 93.66 (2)°
*V* = 1354.3 (4) Å^3^
*Z* = 4
Cu *K*α radiation
μ = 0.77 mm^−1^
*T* = 293 K
0.6 × 0.3 × 0.2 mm

#### Data collection

Oxford Diffraction Xcalibur Ruby diffractometer
Absorption correction: multi-scan (*CrysAlis PRO*; Oxford Diffraction, 2009 ▶) *T*_min_ = 0.699, *T*_max_ = 1.000
5702 measured reflections
2772 independent reflections
1851 reflections with *I* > 2σ(*I*)
*R*_int_ = 0.029

#### Refinement

*R*[*F* ^2^ > 2σ(*F* ^2^)] = 0.042
*wR*(*F* ^2^) = 0.117
*S* = 0.95
2772 reflections
197 parameters
3 restraints
H atoms treated by a mixture of independent and constrained refinement
Δρ_max_ = 0.15 e Å^−3^
Δρ_min_ = −0.17 e Å^−3^

### 

Data collection: *CrysAlis PRO* (Oxford Diffraction, 2009 ▶); cell refinement: *CrysAlis PRO*; data reduction: *CrysAlis PRO*; program(s) used to solve structure: *SHELXS97* (Sheldrick, 2008 ▶); program(s) used to refine structure: *SHELXL97* (Sheldrick, 2008 ▶); molecular graphics: *XP* in *SHELXTL* (Sheldrick, 2008 ▶); software used to prepare material for publication: *publCIF* (Westrip, 2010 ▶).

## Supplementary Material

Crystal structure: contains datablock(s) I, GLOBAL. DOI: 10.1107/S1600536813021028/zq2204sup1.cif

Structure factors: contains datablock(s) I. DOI: 10.1107/S1600536813021028/zq2204Isup2.hkl

Supplementary material file. DOI: 10.1107/S1600536813021028/zq2204Isup3.cml

Additional supplementary materials:  crystallographic information; 3D view; checkCIF report

## Figures and Tables

**Table 1:** Hydrogen-bond geometry (Å, °)

*D*—H⋯*A*	*D*—H	H⋯*A*	*D*⋯*A*	*D*—H⋯*A*
N1—H1⋯O2	0.90 (2)	1.81 (2)	2.608 (2)	146.8 (19)
N1s—H1*A*⋯O1	0.94 (2)	1.89 (2)	2.807 (2)	165 (2)
N1s—H1*B*⋯O3^i^	0.98 (2)	1.76 (2)	2.729 (2)	170 (2)
N1s—H1*C*⋯O2^ii^	0.94 (2)	1.84 (2)	2.753 (2)	163 (2)
C2s—H2*B*⋯O3^iii^	0.97	2.59	3.315 (2)	131
C6—H6⋯O3^iii^	0.93	2.50	3.319 (2)	147
C9—H9⋯O1	0.93	2.25	2.863 (2)	123

Symmetry codes: (i) 
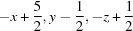
; (ii) 
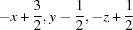
; (iii) 
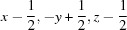
.

**Fig. 1.**
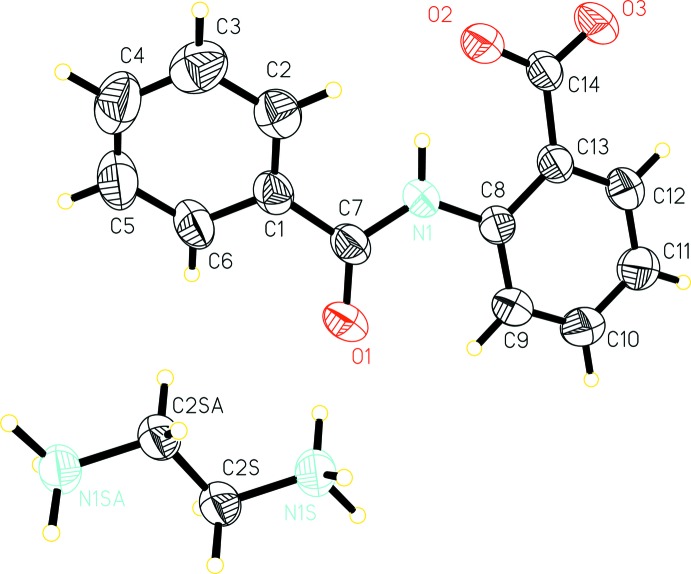
The molecular structure of the title compound with displacement ellipsoids drawn at the 50% probability level.

**Fig. 2.**
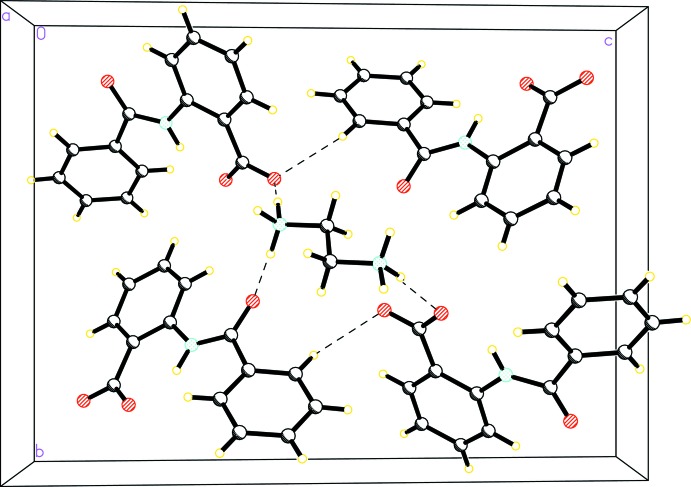
View of the crystal structure along the a axis showing N—H···O hydrogen bonds (dashed lines).

## supplementary crystallographic information

### 1. Comment

The asymmetric unit is composed of one 2-benzamidobenzoate anion and one-half of the ethylenediaminium cation (Fig. 1). In the 2-benzamidobenzoate anion the carboxylate group is slightly rotated with respect to the benzene ring, the dihedral angle between the mean planes is 6.1 (1)°, while the peptide bond exhibits an angle of 6.1 (8)° relative to the benzene ring. The angle between the peptide bond and the second benzene ring is 29.6 (7)°. An intramolecular N—H···O hydrogen bond is observed between the amino group and one O atom of the carboxylate (Table 1). Both amine N atoms of the ethylenediamine are protonated and all nitrogen H atoms are involved in N—H···O hydrogen bonds (Table 1, Fig. 2). In the crystal structure, anions are bridged by diammonium N–H(*b*) and N–H(*c*) bonds *via* the carboxylate O atoms forming centrosymmetrical H-bonded strands along the *a* axis. Other intermolecular N—H···O hydrogen bonds between the diammonium N–H(*a*) bonds and the carbonyl O atoms link these strands into sheets parallel to (011) and (0–11) planes forming a three-dimensional network. In addition, C—H···O weak interactions further stabilize the crystal structure. Some related crystal structures of interest were previously reported by Shen-Tu *et al.* (2008), Zhao and Feng (2011) and Kaizer *et al.* (2006).

### 2. Experimental

A 1:2 mixture of 1,2-ethylenediamine and 2-benzamidobenzoic acid were dissolved in ethanol. Colourless prismatic crystals suitable for a X-ray analysis were obtained after 5 days.

### 3. Refinement

Carbon-bound H atoms were placed geometrically and treated as riding on their parent atoms with C—H = 0.93 Å (aromatic) and 0.97 Å (methylen) with *U*_iso_(H) = 1.2*U*_eq_(C). Nitrogen-bound H atoms, all involved in hydrogen bonds, were located by difference Fourier synthesis and refined isotropically with distance restraints. The refined N–H bonds are in the range of 0.90 (2) - 0.98 (2) Å.

### Crystal data

**Table:**

0.5C_2_H_10_N_2_^2+^·C_14_H_10_NO_3_^−^	*F*(000) = 572
*M**_r_* = 271.29	*D*_x_ = 1.331 Mg m^−^^3^
Monoclinic, *P*2_1_/*n*	Cu *K*α radiation, λ = 1.54184 Å
Hall symbol: -P 2yn	Cell parameters from 753 reflections
*a* = 5.314 (1) Å	θ = 4.0–75.3°
*b* = 13.745 (2) Å	µ = 0.77 mm^−^^1^
*c* = 18.580 (4) Å	*T* = 293 K
β = 93.66 (2)°	Prism, colourless
*V* = 1354.3 (4) Å^3^	0.6 × 0.3 × 0.2 mm
*Z* = 4	

### Data collection

**Table:**

Oxford Diffraction Xcalibur Ruby diffractometer	2772 independent reflections
Radiation source: fine-focus sealed tube	1851 reflections with *I* > 2σ(*I*)
Graphite monochromator	*R*_int_ = 0.029
Detector resolution: 10.2576 pixels mm^-1^	θ_max_ = 75.9°, θ_min_ = 4.0°
ω scans	*h* = −5→6
Absorption correction: multi-scan (*CrysAlis PRO*; Oxford Diffraction, 2009)	*k* = −16→16
*T*_min_ = 0.699, *T*_max_ = 1.000	*l* = −23→23
5702 measured reflections	

### Refinement

**Table:**

Refinement on *F*^2^	Primary atom site location: structure-invariant direct methods
Least-squares matrix: full	Secondary atom site location: difference Fourier map
*R*[*F*^2^ > 2σ(*F*^2^)] = 0.042	Hydrogen site location: inferred from neighbouring sites
*wR*(*F*^2^) = 0.117	H atoms treated by a mixture of independent and constrained refinement
*S* = 0.95	*w* = 1/[σ^2^(*F*_o_^2^) + (0.068*P*)^2^] where *P* = (*F*_o_^2^ + 2*F*_c_^2^)/3
2772 reflections	(Δ/σ)_max_ < 0.001
197 parameters	Δρ_max_ = 0.15 e Å^−^^3^
3 restraints	Δρ_min_ = −0.17 e Å^−^^3^

### Special details

**Table:**

Geometry. All e.s.d.'s (except the e.s.d. in the dihedral angle between two l.s. planes) are estimated using the full covariance matrix. The cell e.s.d.'s are taken into account individually in the estimation of e.s.d.'s in distances, angles and torsion angles; correlations between e.s.d.'s in cell parameters are only used when they are defined by crystal symmetry. An approximate (isotropic) treatment of cell e.s.d.'s is used for estimating e.s.d.'s involving l.s. planes.
Refinement. Refinement of *F*^2^ against ALL reflections. The weighted *R*-factor *wR* and goodness of fit *S* are based on *F*^2^, conventional *R*-factors *R* are based on *F*, with *F* set to zero for negative *F*^2^. The threshold expression of *F*^2^ > σ(*F*^2^) is used only for calculating *R*-factors(gt) *etc*. and is not relevant to the choice of reflections for refinement. *R*-factors based on *F*^2^ are statistically about twice as large as those based on *F*, and *R*- factors based on ALL data will be even larger.

### Fractional atomic coordinates and isotropic or equivalent isotropic displacement parameters (Å^2^)

**Table:**

	*x*	*y*	*z*	*U*_iso_*/*U*_eq_	
O1	0.9888 (3)	0.12659 (9)	0.12307 (7)	0.0596 (4)	
O2	1.0308 (2)	0.35350 (9)	0.32469 (7)	0.0589 (4)	
O3	1.3389 (2)	0.35553 (9)	0.41015 (6)	0.0563 (4)	
N1	1.0384 (3)	0.22290 (11)	0.22300 (7)	0.0441 (3)	
H1	0.990 (4)	0.2773 (15)	0.2457 (11)	0.069 (6)*	
C1	0.7412 (3)	0.27034 (13)	0.12662 (9)	0.0441 (4)	
C2	0.5906 (3)	0.32616 (14)	0.16836 (10)	0.0528 (5)	
H2	0.6089	0.3221	0.2184	0.063*	
C3	0.4114 (4)	0.38855 (16)	0.13553 (13)	0.0678 (6)	
H3	0.3084	0.4256	0.1635	0.081*	
C4	0.3873 (4)	0.39519 (17)	0.06148 (14)	0.0761 (7)	
H4	0.2667	0.4364	0.0394	0.091*	
C5	0.5398 (5)	0.34150 (18)	0.02018 (12)	0.0758 (7)	
H5	0.5256	0.3476	−0.0298	0.091*	
C6	0.7144 (4)	0.27842 (15)	0.05216 (10)	0.0592 (5)	
H6	0.8148	0.2410	0.0237	0.071*	
C7	0.9329 (3)	0.19958 (13)	0.15740 (8)	0.0440 (4)	
C8	1.2370 (3)	0.17703 (12)	0.26353 (8)	0.0407 (4)	
C9	1.3484 (4)	0.09159 (13)	0.24163 (9)	0.0517 (4)	
H9	1.2873	0.0611	0.1994	0.062*	
C10	1.5489 (4)	0.05158 (14)	0.28199 (10)	0.0582 (5)	
H10	1.6229	−0.0054	0.2665	0.070*	
C11	1.6410 (4)	0.09508 (15)	0.34524 (11)	0.0609 (5)	
H11	1.7753	0.0676	0.3726	0.073*	
C12	1.5309 (3)	0.17992 (14)	0.36727 (9)	0.0528 (5)	
H12	1.5931	0.2092	0.4099	0.063*	
C13	1.3301 (3)	0.22301 (12)	0.32778 (8)	0.0405 (4)	
C14	1.2281 (3)	0.31706 (13)	0.35585 (8)	0.0431 (4)	
N1S	0.7225 (3)	−0.04084 (12)	0.07687 (8)	0.0487 (4)	
H1A	0.784 (4)	0.0205 (12)	0.0916 (10)	0.074 (7)*	
H1B	0.869 (4)	−0.0844 (15)	0.0810 (12)	0.086 (7)*	
H1C	0.612 (4)	−0.0677 (16)	0.1087 (10)	0.081 (7)*	
C2S	0.6042 (3)	−0.03697 (13)	0.00242 (8)	0.0455 (4)	
H2A	0.5369	−0.1005	−0.0110	0.055*	
H2B	0.7303	−0.0199	−0.0309	0.055*	

### Atomic displacement parameters (Å^2^)

**Table:**

	*U*^11^	*U*^22^	*U*^33^	*U*^12^	*U*^13^	*U*^23^
O1	0.0732 (8)	0.0542 (8)	0.0492 (7)	0.0034 (7)	−0.0132 (6)	−0.0188 (6)
O2	0.0714 (9)	0.0551 (8)	0.0482 (7)	0.0153 (7)	−0.0127 (6)	−0.0139 (6)
O3	0.0657 (8)	0.0587 (8)	0.0432 (6)	−0.0028 (7)	−0.0074 (5)	−0.0163 (6)
N1	0.0558 (8)	0.0405 (8)	0.0349 (7)	0.0005 (7)	−0.0062 (6)	−0.0052 (6)
C1	0.0458 (9)	0.0437 (9)	0.0417 (8)	−0.0107 (7)	−0.0059 (7)	−0.0015 (7)
C2	0.0517 (10)	0.0550 (11)	0.0515 (10)	−0.0089 (9)	0.0014 (8)	−0.0004 (9)
C3	0.0528 (11)	0.0610 (13)	0.0891 (16)	0.0023 (10)	0.0010 (10)	−0.0037 (12)
C4	0.0657 (13)	0.0706 (15)	0.0881 (17)	0.0003 (12)	−0.0257 (12)	0.0134 (13)
C5	0.0872 (16)	0.0810 (16)	0.0556 (12)	0.0008 (14)	−0.0229 (11)	0.0081 (11)
C6	0.0674 (12)	0.0656 (12)	0.0425 (9)	−0.0035 (10)	−0.0127 (8)	−0.0046 (9)
C7	0.0515 (9)	0.0440 (9)	0.0359 (8)	−0.0081 (8)	−0.0017 (7)	−0.0048 (7)
C8	0.0482 (9)	0.0382 (8)	0.0352 (8)	−0.0022 (7)	−0.0009 (6)	0.0007 (7)
C9	0.0648 (11)	0.0439 (9)	0.0454 (9)	0.0003 (9)	−0.0052 (8)	−0.0072 (8)
C10	0.0667 (12)	0.0472 (10)	0.0599 (11)	0.0112 (9)	−0.0026 (9)	−0.0058 (9)
C11	0.0630 (12)	0.0580 (12)	0.0595 (11)	0.0127 (10)	−0.0142 (9)	−0.0045 (10)
C12	0.0580 (11)	0.0560 (11)	0.0426 (9)	0.0020 (9)	−0.0108 (8)	−0.0054 (8)
C13	0.0469 (9)	0.0403 (9)	0.0341 (8)	−0.0035 (7)	0.0008 (6)	−0.0004 (7)
C14	0.0528 (9)	0.0442 (9)	0.0320 (7)	−0.0032 (8)	0.0006 (6)	−0.0015 (7)
N1S	0.0525 (9)	0.0487 (9)	0.0438 (8)	−0.0061 (8)	−0.0055 (7)	0.0056 (7)
C2S	0.0531 (10)	0.0462 (9)	0.0368 (8)	−0.0016 (8)	0.0000 (7)	0.0001 (7)

### Geometric parameters (Å, º)

**Table:**

O1—C7	1.235 (2)	C8—C9	1.388 (2)
O2—C14	1.268 (2)	C8—C13	1.412 (2)
O3—C14	1.2523 (18)	C9—C10	1.377 (2)
N1—C7	1.3474 (19)	C9—H9	0.9300
N1—C8	1.406 (2)	C10—C11	1.380 (2)
N1—H1	0.90 (2)	C10—H10	0.9300
C1—C2	1.382 (3)	C11—C12	1.378 (3)
C1—C6	1.386 (2)	C11—H11	0.9300
C1—C7	1.495 (2)	C12—C13	1.388 (2)
C2—C3	1.393 (3)	C12—H12	0.9300
C2—H2	0.9300	C13—C14	1.508 (2)
C3—C4	1.377 (3)	N1S—C2S	1.483 (2)
C3—H3	0.9300	N1S—H1A	0.940 (15)
C4—C5	1.367 (3)	N1S—H1B	0.983 (16)
C4—H4	0.9300	N1S—H1C	0.936 (15)
C5—C6	1.376 (3)	C2S—C2S^i^	1.501 (3)
C5—H5	0.9300	C2S—H2A	0.9700
C6—H6	0.9300	C2S—H2B	0.9700
			
C7—N1—C8	129.24 (16)	C8—C9—H9	119.7
C7—N1—H1	120.3 (13)	C9—C10—C11	120.70 (18)
C8—N1—H1	110.4 (13)	C9—C10—H10	119.7
C2—C1—C6	119.29 (18)	C11—C10—H10	119.7
C2—C1—C7	123.45 (15)	C12—C11—C10	119.03 (17)
C6—C1—C7	117.25 (17)	C12—C11—H11	120.5
C1—C2—C3	120.02 (18)	C10—C11—H11	120.5
C1—C2—H2	120.0	C11—C12—C13	122.03 (16)
C3—C2—H2	120.0	C11—C12—H12	119.0
C4—C3—C2	119.7 (2)	C13—C12—H12	119.0
C4—C3—H3	120.1	C12—C13—C8	118.15 (16)
C2—C3—H3	120.1	C12—C13—C14	117.66 (14)
C5—C4—C3	120.3 (2)	C8—C13—C14	124.19 (14)
C5—C4—H4	119.9	O3—C14—O2	122.25 (16)
C3—C4—H4	119.9	O3—C14—C13	118.66 (15)
C4—C5—C6	120.3 (2)	O2—C14—C13	119.07 (14)
C4—C5—H5	119.8	C2S—N1S—H1A	111.1 (12)
C6—C5—H5	119.8	C2S—N1S—H1B	112.5 (14)
C5—C6—C1	120.3 (2)	H1A—N1S—H1B	105.2 (18)
C5—C6—H6	119.8	C2S—N1S—H1C	110.9 (13)
C1—C6—H6	119.8	H1A—N1S—H1C	113.1 (19)
O1—C7—N1	124.08 (17)	H1B—N1S—H1C	103.8 (19)
O1—C7—C1	120.75 (14)	N1S—C2S—C2S^i^	110.32 (17)
N1—C7—C1	115.16 (15)	N1S—C2S—H2A	109.6
C9—C8—N1	122.83 (15)	C2S^i^—C2S—H2A	109.6
C9—C8—C13	119.58 (15)	N1S—C2S—H2B	109.6
N1—C8—C13	117.55 (15)	C2S^i^—C2S—H2B	109.6
C10—C9—C8	120.52 (16)	H2A—C2S—H2B	108.1
C10—C9—H9	119.7		

Symmetry code: (i) −*x*+1, −*y*, −*z*.

### Hydrogen-bond geometry (Å, º)

**Table:**

*D*—H···*A*	*D*—H	H···*A*	*D*···*A*	*D*—H···*A*
N1—H1···O2	0.90 (2)	1.81 (2)	2.608 (2)	146.8 (19)
N1s—H1*A*···O1	0.94 (2)	1.89 (2)	2.807 (2)	165 (2)
N1s—H1*B*···O3^ii^	0.98 (2)	1.76 (2)	2.729 (2)	170 (2)
N1s—H1*C*···O2^iii^	0.94 (2)	1.84 (2)	2.753 (2)	163 (2)
C2s—H2*B*···O3^iv^	0.97	2.59	3.315 (2)	131
C6—H6···O3^iv^	0.93	2.50	3.319 (2)	147
C9—H9···O1	0.93	2.25	2.863 (2)	123
C12—H12···O3	0.93	2.42	2.758 (2)	101

Symmetry codes: (ii) −*x*+5/2, *y*−1/2, −*z*+1/2; (iii) −*x*+3/2, *y*−1/2, −*z*+1/2; (iv) *x*−1/2, −*y*+1/2, *z*−1/2.
